# Topical Umbilical Cord Serum for Corneal Epithelial Defects after Diabetic Vitrectomy

**DOI:** 10.18502/jovr.v15i2.6732

**Published:** 2020-04-06

**Authors:** Siamak Moradian, Marzieh Ebrahimi, Azade Kanaani, Amir Faramarzi, Sare Safi

**Affiliations:** ^1^ Ophthalmic Epidemiology Research Center, Shahid Beheshti University of Medical Sciences, Tehran, Iran; ^2^ Ophthalmic Research Center, Shahid Beheshti University of Medical Sciences, Tehran, Iran; ^3^ Department of Stem Cells, Cell Science Research Center, Royan Institute, Tehran, Iran

**Keywords:** Corneal Epithelial Defect, Diabetic Vitrectomy, Topical Umbilical Cord Serum

## Abstract

**Purpose:**

To evaluate the role of topical umbilical cord serum (TUCS) therapy in treating corneal epithelial defects (CEDs) after diabetic vitrectomy.

**Methods:**

In this double-masked, randomized clinical trial, we included 80 eyes of 80 patients who were candidates for vitrectomy due to proliferative diabetic retinopathy complications. In cases of corneal edema obscuring the fundus view during surgery, the corneal epithelium was removed using a 6-mm trephine and a blade no.15. The day after the surgery, patients were randomly divided into two groups: (1) the TUCS group that received 20% TUCS six times/day in addition to the conventional treatment of CED and (2) the control group, which was prescribed artificial tears as placebo in addition to the conventional treatment of CED. The rate of healing of CEDs was measured via two maximum linear dimensions perpendicular to each other at the start of therapy and on postoperative days 1–5, 7, and 12.

**Results:**

Of 80 eyes, 40 were assigned to each treatment group. The mean times to complete CED healing were 2.4 ± 0.7 and 3.8 ± 2.1 days in the TUCS and control groups, respectively (*P* < 0.001). Persistent CED occurred in two eyes in the control group but in no eyes in the TUCS group.

**Conclusion:**

TUCS therapy may be safe and effective in healing CEDs after vitrectomy in patients with diabetes.

##  INTRODUCTION

The removal of corneal epithelium may be necessary to improve a surgeon's visualization of fundus during vitrectomy. In diabetic patients, abnormal postoperative healing of corneal epithelial defects (CEDs) results in morbidity and prolongs the hospitalization period. Generally, patients with diabetes are at a high risk of developing corneal disorders, a condition known as diabetic keratopathy due to insufficient epithelial adherence to the Bowman's membrane. Significant recurrent corneal erosions can occur after intraocular surgery, particularly vitrectomy, in such patients.^[1,2]^ Treatment options for diabetic keratopathy are limited. Recent studies have reported the effectiveness of topical umbilical cord serum (TUCS) in treating ocular surface disorders, such as neurotrophic keratitis, dry eye syndrome, and persistent CEDs.^[3,4,5]^ Several growth factors, such as the epidermal growth factor (EGF), acidic and basic fibroblast growth factors (FGFs), platelet-derived growth factor, hepatocyte growth factor, vitamin A, transforming growth factor-β (TGF- β), substance P, insulin-like growth factor-1 (IGF-1), nerve growth factor (NGF), fibronectin, and α ${}_{2}$ -macroglobulin, are present in umbilical cord serum (UCS). Furthermore, concentrations of EGF, TGF-B, and NGF are several times higher in UCS than in peripheral blood.^[4,5]^ We performed this study to investigate whether TUCS may have a beneficial role in healing CEDs after diabetic vitrectomy.

##  METHODS

In this double-masked, prospective randomized controlled clinical trial (RCT), we included all patients with type II diabetes who underwent deep vitrectomy because of proliferative diabetic retinopathy (PDR) complications and had corneal epithelial edema during surgery that prompted removal of the corneal epithelium to improve the media clarity. A 6-mm trephine was used to equalize the basic CED size.

The exclusion criteria were a history of herpetic, exposure, or neurotrophic keratitis, previous keratorefractive surgery, significant dry eye, trachomatous keratopathy, immune or nutritional deficiency, a history of autoimmune diseases, lid abnormality, use of glaucoma eye drops or any other eye drop after surgery (except for drops that were used during the study), and pregnancy. Eyes with severe lid edema after surgery were also excluded.

The study protocol was approved by the institutional review board of the Ophthalmic Research Center of the Shahid Beheshti University of Medical Sciences. This study was conducted according to the Declaration of Helsinki and all participants gave written informed consent before entering the study. This RCT was registered at www.ClinicalTrial.gov (NCT01168375).

Patients in the TUCS group received 20% TUCS eye drops (diluted with preservative-free artificial tears) six times/day in addition to the conventional treatment (chloramphenicol and betamethasone eye drops four times daily and cycloplegic eye drops three times daily). Patients in the control group received the conventional treatment and a placebo (preservative-free artificial tears), without patching the eye between the drop administrations.

Patients were followed-up on postoperative days 1–5, 7, and 12. Corneal photography was performed after administration of the fluorescein dye. The longest linear diameter and the longest vertical diameter of the CED area were measured and multiplied to obtain the area of the equivalent rectangle. The rate of healing of CEDs was measured at each subsequent follow-up. The umbilical cord blood was obtained from healthy mothers with uncomplicated cesarean section delivery after screening for parenterally transmitted viral diseases, hepatitis B and C, human immunodeficiency virus, cytomegalovirus, and syphilis at the Royan Institute Cord Blood Bank. Briefly, samples that were negative for viruses and microbes were centrifuged at 1,500 rotations/min for 5 min, and the supernatant cells were discarded. No anticoagulants were used in the procedure. Umbilical cord serum was stored at –20.0°C in 5 ml bottles. The maximum storage time was three months. To prepare eye drops, one bottle was opened, and UCS was diluted with preservative-free artificial tears to achieve the 20% concentration. Samples were then stored at 4.0°C for three days. Subsequently, a new bottle was opened, and the old bottle was discarded. Randomization was performed using the random-block permutation method using a computer-generated randomized list. Patients were then assigned to one of the two groups: case or control. A random allocation sequence was performed by a biostatistician. Details of the series were unknown to the study investigators. All study personnel and participants were masked to the treatment throughout the study. TUCS and placebo eye drops were prepared and labeled using codes by a non-study ward staff based on the randomization list provided. Only the study biostatistician who had no contact with the study participants was unmasked to the treatment groups.

### Sample Size

In the pilot study, the standard deviations of time to remission in the umbilical cord serum and placebo groups were 0.8 and 2.0, respectively. To achieve an 85% power in detecting one-day differences between the new intervention and conventional treatment groups with a type-I error of 5%, we needed 40 patients in each group.

### Statistical Analysis 

Data are expressed as mean ± standard deviation, median, range, percent, and 95% confidence interval (CI). To evaluate differences between groups, we used the Chi-square test, Fisher's exact test and *t*-test, whenever appropriate. Relationships between age and duration of operation and time to remission were evaluated using Spearman's correlation coefficient. Analyses were performed using SPSS (version 17.0, SPSS Inc., Chicago, IL, USA). *P* < 0.05 was considered statistically significant.

##  RESULTS

In total, 80 eyes of 80 patients (42 women [52.5%] and 38 men [47.5%]), with a mean age of 58.5 ± 10.3 (median: 58; range: 34 to 80) years, were enrolled in this study. Their mean basic CED size was 15.5 ± 6.5 (median: 14; range: 2 to 33) mm ${}^{2}$ the day after the surgery [Table 1].

**Figure 1:**
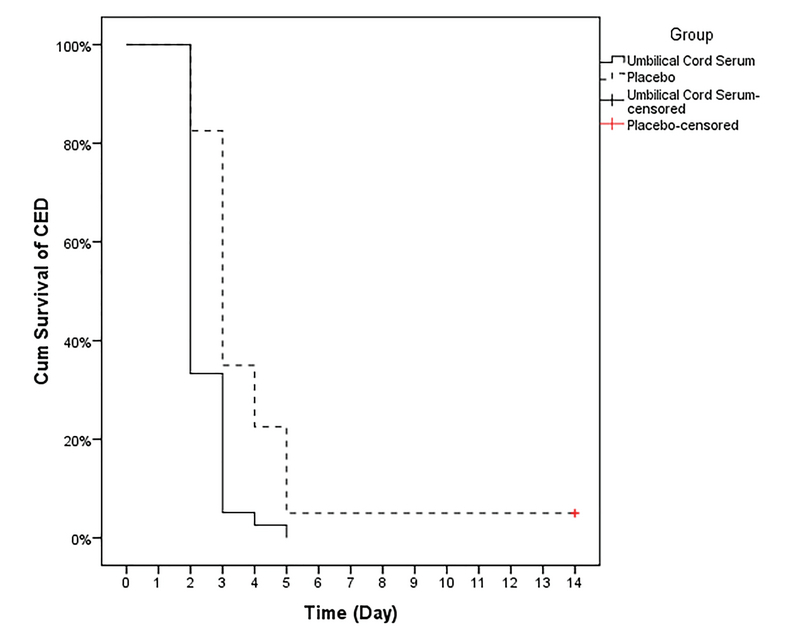
Rate of corneal epithelial defect improvement in the topical umbilical cord serum and control groups. CED, corneal epithelial defect; Cum, cumulative

**Table 1:** Baseline characteristics of participants


**Variable**	**Statistics**	**Groups**		***P*** **-value**
	**Umbilical cord serum (** ***n*** ** = 40)**	**Placebo (** ***n*** ** = 40)**	
Age (years)	Mean ± SD (Range)	57.6 ± 9.6 (34 to 78)	59.5 ± 11.0 (37 to 80)	0.400 ${}^{†}$
Sex	F/M (F%)	22/55 (55%)	20/20 (50%)	0.654*
Basic CED Size	Mean ± SD (Range)	15.3 ± 6.2 (2.3 to 30)	15.7 ± 6.8 (2 to 33)	0.801 ${}^{†}$
Operation duration (hours)	Mean ± SD (Range)	1.65 ± 0.62 (1 to 4)	1.45 ± 0.50 (1 to 2)	0.118 ${}^{**}$
${}^{†}$ Based on t-test; ${}^{*}$ Based on Chi-square test; ${}^{**}$ based on Fisher’s exact test. CED, corneal epithelial defect; F, female; M, male; SD, standard deviation			

**Table 2:** Mean ± standard deviation (range) of the rate of improvement (days)


**Operation**	**Groups**			
	**Umbilical cord serum (** ***n*** ** = 40)**	**Placebo (** ***n*** ** = 40)**	**Total**	**Diff (95% CI)**	***P*** **-value (Between)**
Vitrectomy	2.4 ± 0.7 (2 to 5)	3.8 ± 2.1 (2 to 12)	–1.3 (–2.1 to –0.6)	< 0.001 †
† Based on t-test					

**Table 3:** Correlation of age and duration of operation in each group with the rate of improvement of corneal epithelial defects


	**Groups**				
	**Umbilical cord serum (** ***n*** ** = 40)**		**Controls (** ***n*** ** = 40)**		**Total**	
	**Correlation**	***P*** **-value**	**Correlation**	***P*** **-value**	**Correlation**	***P*** **-value**
Age	0.205	0.211	–0.004	0.979	0.073	0.522
Operation duration	–0.202	0.219	–0.202	0.212	–0.218	0.054
Based on Spearman’s correlation coefficient					

As demonstrated in Table 2 and Figure 1, the rate of improvement of CEDs was 1.3 days lesser in the case group than in the control group (95% CI: 0.6–2.1, *P* < 0.001).

There was no statistically significant difference between men and women in the rate of improvement (3.13 ± 1.74 in men vs 3.05 ± 1.73 in women, *P* = 0.833) in the case and control groups (*P* = 0.451 and *P* = 0.885, respectively).

Additionally, we found no significant correlation of the age or duration of operation with the overall rate of improvement in either group [Table 3]. Two cases of persistent CED occurred in the control group, but no eyes in the TUCS group showed this complication.

##  DISCUSSION

Diabetic vitrectomy typically necessitates pars plana vitrectomy to treat complications of PDR. Corneal epithelial edema and late corneal decompensation can occur during vitreoretinal surgery.^[6]^ Intraoperative corneal epithelial edema causes media haziness; therefore, the surgeon may mechanically scrape the epithelium with a blade to improve the media clarity and visualization of the fundus. Intraoperative removal of the corneal epithelium or accidental surgical trauma to the corneal epithelium results in postoperative CED, and healing of such lesions can be delayed, particularly in patients with diabetes.^[2,7]^ Postoperative CED is a significant cause of morbidity in these patients. Virata et al reported that 45% of such cases of CEDs persisted for longer than one week and 28% lasted for four or more weeks. One reported case resulted in a bacterial corneal ulcer.^[8]^ In one study, the overall rate of corneal epithelial debridement during vitrectomy averaged among 55 vitreoretinal surgeons was 17.4% ± 19.0% and ranged from 0% to 90%,^[9]^ although other studies reported this rate to be approximately 14–34%.^[10,11]^ Both structural (polymegathism and pleomorphism) and functional abnormalities (increased permeability and slower recovery from induced edema) are observed in the diabetic corneal epithelium and endothelium.^[12]^ EGF,^[13,14,15,16]^ FGF, and interlukin-6^[17]^ stimulate corneal epithelial migration and proliferation. Fibronectin,^[18]^ hyaluronan,^[19]^ laminin,^[20]^ and collagen type IV are the extracellular matrix components that can facilitate epithelial cell migration. Neuronotrophic substances, such as substance P, IGF-I, and NGF promote corneal epithelial migration; topical application promotes corneal wound healing.^[21,22,23,24,25]^ EGF, vitamin A, substance P, α ${}_{2}$ -macroglobulin, fibronectin, and acidic and basic FGFs are tear components that play major roles in proliferation, differentiation, and maturation of the corneal epithelial cells.^[26,27]^ Autologous serum contains factors, such as glucose, proteins, calcium ion, vitamin A, EGF, fibronectin, and glycoproteins, that facilitate corneal wound healing.^[28,29,30]^ Therefore, autologous serum eye drops are used to treat ocular surface disorders.^[31]^

Autologous serum eye drops are effective in cases of keratoconjunctivitis sicca,^[32]^ severe dry eye,^[33,34]^ superior limbal keratoconjunctivitis,^[35]^ persistent CED, and neurotrophic keratopathies.^[36]^ Higher concentrations of EGF, vitamin A, acidic and basic FGFs, fibronectin, NGF, substance P, and α ${}_{2}$ -macroglobulin, which are present in UCS, make it more effective than autologous serum in treating ocular surface disorders.^[31]^ Recently, Vajpayee et al reported that TUCS eye drops resulted in faster healing of persistent CED refractory to medical treatments compared to autologous serum drops.^[37]^ Yoon et al found that the application of TUCS effectively treated dry eye and persistent CED due to neurotrophic keratitis.^[3,4,5]^ Autologous serum preparation requires collection of blood at regular intervals from patients and may induce discomfort. It is difficult to obtain blood samples from patients with a poor general condition or blood dyscrasia, but abundant serum can be drawn from the umbilical vein at once. Many patients can benefit from this sampling approach, which minimizes discomfort.^[5]^ In this study, we examined the effects of TUCS therapy on improvement of CED after diabetic vitrectomy. Our study showed that TUCS hastened the healing of CEDs after vitrectomy relative to the conventional therapy. Persistent CED occurred in two cases in the control group but in no cases in the TUCS group.

The limitations of our study were the absence of dry eye tests before surgery and unavailability of precise measurement of CEDs using Image J or other digital measurement modalities.

In conclusion, TUCS may significantly accelerate the rate of improvement of CEDs after diabetic vitrectomy and reduce the risk of persistent CED. Further studies with larger sample size are needed.

##  Financial Support and Sponsorship

None.

##  Conflicts of Interest

There are no conflicts of interest.
